# Prevalence of Symptomatic Reherniation After Lumbar Discectomy Using a Bone-Anchored Annular Closure Device and Associated Contributing Factors: A Meta-Analysis

**DOI:** 10.5704/MOJ.2603.007

**Published:** 2026-03

**Authors:** ST Al-Gunaid, M Iqhrammullah, G Maulana, I Qanita, MA Adista, I Hidayat

**Affiliations:** 1 Department of Medicine, Universiti Syiah Kuala, Banda Aceh, Indonesia; 2 Department of Public Health, Universitas Muhammadiyah Aceh, Banda Aceh, Indonesia; 3 Department of Neurology, Universiti Syiah Kuala, Banda Aceh, Indonesia; 4 Department of Surgery, Universiti Syiah Kuala, Banda Aceh, Indonesia

**Keywords:** bone-anchored annular closure device, ACD, recurrent disc herniation, lumbar discectomy, prevalence

## Abstract

**Introduction:**

The primary issue following lumbar discectomy for disc herniation is the risk of reherniation in the post-operative period. Many surgical techniques have been proposed to treat disc reherniation, however, the optimal one remains variable. This meta-analysis aimed to investigate the prevalence of symptomatic reherniation after using a Bone-anchored annular closure device following lumbar discectomy and the contributing factors.

**Materials and methods:**

Identification of published literature was performed on PubMed, Google Scholar, Scopus, and Web of Science databases. Studies published until 14 February 2024 reported the prevalence of symptomatic reherniation after using a Bone-anchored annular closure device following lumbar discectomy and the associated contributing factors. A random effects model was used to conduct Bayesian frequentist network meta-analysis and pair-wise meta-analysis, with the assessment based on standardised mean difference (SMD) and 95% confidence interval (CI).

**Results:**

Eleven studies published in 2012 − 2022 recruiting a total of 5195 patients were included in the meta-analysis. The prevalence of reherniation in ACD and control groups was 23.2% (95% CI: 18.2% − 28.1%) and 36.4% (95% CI: 28.2% − 44.5%), respectively. The moderator effect of sample size is significant for pooled data of the ACD group (p-mod=0.002), but not for the control group (p-mod=0.278). After the adjustment with sample size, the prevalence rates were 13.6% (95% CI: 6.2% − 21.1%) and 29.6% (95% CI: 14.9% − 33.2%) for ACD and control groups, respectively.

**Conclusion:**

Comparatively to lumbar discectomy alone, using a Bone-anchored annular closure device following lumbar discectomy decreased the symptomatic reherniation rate and post-operative complications, as well as the necessity for subsequent surgeries.

## INTRODUCTION

Lumbar herniation occurs most frequently in adult population, affecting around 29% of individuals at 20 years old and increasing to as much as 43% at 80 years old^1^. The primary symptoms of herniation include pain radiating from the buttocks and down the leg, stemming from the distribution of the lumbar nerve root. Conservative treatments such as physical therapy, pharmacological therapy, and steroid injection are often sufficient to improve the condition of most patients with lumbar herniation. However, some individuals may experience persistent radiating pain with neurological deficits, leading to the consideration of surgical interventions^2^.

Lumbar discectomy is a commonly performed procedure for intervertebral disc herniation^3^. Even though lumbar discectomy is generally considered to be safe and provides initial relief from symptoms for most patients, the risk of perioperative complications falls within the range of 13% to 15%^4-6^. Patients with large annular defects after lumbar discectomy have a higher likelihood of recurrent disc herniation during long-term follow-up^7,8^. Therefore, it is likely that these patients may face a greater risk of complications during the perioperative period^5^. Perioperative complications result in significant burdens for patients, healthcare providers, and hospitals^9^.

Reducing complication rates, whether during hospitalisation or after discharge, is important^10^. Implementing treatment pathways that lower the risk of perioperative complications following lumbar discectomy could enhance patient outcomes and reduce healthcare expenses^9,11^. A newly developed approach involving the use of the Annular Closure Device (ACD) has been employed to prevent reherniation in patients who have undergone surgery. ACD is utilised to obstruct a broader annular defect and maintain the nucleus pulposus within the disc space^8,12,13^. Numerous studies demonstrated that ACD implantation, when combined with lumbar discectomy, resulted in a low probability of recurrent herniation and symptoms thereafter^5,8,9^. Therefore, this systematic review and meta-analysis aimed to investigate the prevalence of symptomatic reherniation after using a Bone-anchored annular closure device following lumbar discectomy and the contributing factors.

## MATERIALS AND METHODS

Prior to conducting the review, we created a series of protocols following the PRISMA (Preferred Reporting Items for Systematic Reviews and Meta-Analyses) guidelines. Our research question was centred around evaluating the prevalence of symptomatic reherniation following ACD and the associated factors.

A literature search was conducted on 14 February 2024, using search engines such as PubMed, Google Scholar, Scopus, and Web of Science (WoS). Boolean operators ‘AND’ and ‘OR’ were utilised to form the following combination: [(bone-anchored annular closure device) OR ACD OR (annular closure device) OR (annular device) OR (annular repair) OR (annulus device)] AND [(disc herniation) OR (recurrent disc herniation) OR (prolapsed disc) OR (herniated disc) OR (disc displacement) OR (disc prolapse) OR (prolapsed disk) OR (herniated disk) OR (disk displacement) OR (disk prolapse)]. The search terms were adjusted accordingly in each database.

Studies reporting the symptomatic reherniation rate after using a Bone-anchored annular closure device following lumbar discectomy were included. Articles that provided a comparative study between a population that underwent the additional procedure (ACD) compared to a control group were also included. Eligible study designs were cross-sectional and cohort studies, while case-control studies, case series, review articles, editorials, commentaries, and conference abstracts were excluded.

Following the automatic removal of duplicate entries in EndNote 19, the screening process involved two stages. The first phase involved reviewing the abstract and title, while the second phase involved reviewing the full text. Both stages were conducted separately by two review authors (S.A. and G.M.), and any differences were resolved through consensus. If consensus could not be reached, the third review author (M.I.) was consulted.

The quality of the included studies was evaluated independently by two review authors (S.A. and G.M.). The Newcastle-Ottawa Scale (NOS) was used for assessing observational studies, with a detailed description of this tool having been provided previously^14^. Any disagreements were resolved through consensus or by consulting the third review author (M.I.).

An analysis of the included studies was initiated by extracting their characteristics, including the name of the first author, year of publication, research design, and sample size. Demographic data of the research subjects was then extracted, including age, gender, BMI, and smoking status. Clinical characteristics included level of herniation and type of reherniation (symptomatic reherniation, asymptomatic reherniation). Mean ± standard deviation (SD) was used for presenting continuous data; otherwise, conversion was made using the suggested methods from a previous study^15^. Data extraction was initially carried out by (S.A) and then reconfirmed by the second review author (G.M.).

The prevalence was calculated by multiplying 100% by the pooled proportion of reherniation events and total sample. To calculate the pooled proportion, data were first transformed using the Freeman-Tukey double arcsine method, followed by estimation under restricted maximum likelihood. The calculation also computed a 95% confidence interval (CI). The moderator effect was used to adjust the pooled proportion using several variables such as sample size, mean age, and BMI. The difference in prevalence between ACD and control groups was assessed based on Z-statistics. Additionally, the risk of reherniation was measured by the odds ratio (OR) by comparing the event rate in ACD to that in control groups through a pooled analysis using the restricted maximum-likelihood model. The risk was considered significant if OR > 1 and p-tot < 0.05. The pooled estimate was considered heterogeneous if I^2^> 50% or p-Het < 0.1. Publication bias was identified through a funnel plot, with asymmetry assessed using a rank correlation test (p-Begg’s < 0.05). Pooled analysis was performed using the open-source desktop software Jamovi 2.3.28.0.

## RESULTS

**Fig. 1:**
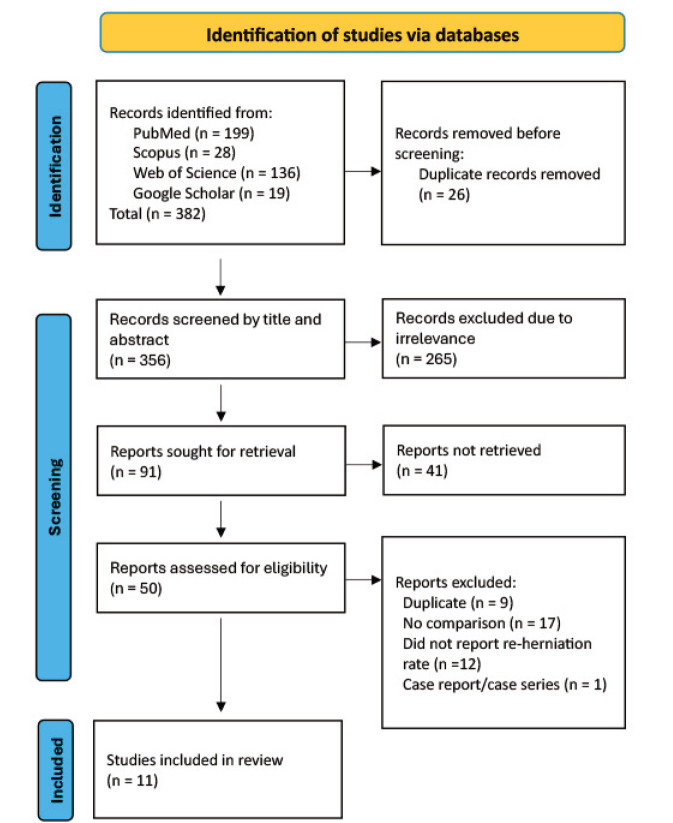
PRISMA flow-chart for the screening and selection of studies reporting the prevalence of symptomatic reherniation.

The initial screening of four databases resulted in the identification of 382 records. With duplicates removed, 356 records remained for abstract and title screening. As many as 91 records were sought for full-text, but only 55 of them had accessible full-text. We identified 9 duplicate records, 17 records that lacked a comparison, 12 records that did not report reherniation rates, and 1 record that was excluded due to being a case report or case series. Finally, we determined that 11 studies were eligible for qualitative and quantitative reviews. The PRISMA flow diagram depicting the overall process of the screening and selection is presented in Fig. 1.

The key features of the studied research are shown in Table I^4-6,8-13,16-29^. The twenty-three articles that made up this systematic review discuss the prevalence of symptomatic reherniation after using a Bone-anchored annular closure device following lumbar discectomy and the contributing factors. The twenty-three investigations were published in English and were conducted in different parts of the world. Furthermore, the use of a Bone-anchored annular closure device following lumbar discectomy was discussed in all twenty-three articles. A total of 5,195 subjects made up the entire sample size across the twenty-three investigations. The distinctive features of each study are presented across the rows, while the general research attributes of each column are given beneath it.

**Table I:** Characteristics of the included studies.

**Author, Year**	**Country**	**Study design**	**Characteristics**		
			**Variable**	**ACD**	**Non-ACD**
Pyung *et al* (2019)^4^	South Korea	Cohort	n Age	30 41.37	30 42.63
			Sex (M / F)%	66.7 / 33.3	83.3 / 16.7
			BMI	24.41	24.63
Claudius *et al* (2020)^5^	Austria, Belgium, Germany, Netherland, Switzerland, France	Cohort	n Age Sex (M / F)%	272 43 57 / 43	Not applicable 38 62 / 38
Michiel *et al* (2012)^6^	Not provided	Cohort	BMI n Age Sex (M / F)% BMI	26 45 42.3 53.3 / 46.7 26.0	26 Not applicable Not applicable Not applicable Not applicable
Adisa *et al* (2017)^8^	Germany	Cohort	n Age Sex (M / F)% BMI	171 45 56.7 / 43.3 Not applicable	Not applicable Not applicable Not applicable Not applicable
Peter *et al* (2017)^9^	Germany, Belgium, Switzerland, France, Austria Netherlands	Cohort	n Age Sex (M / F)% BMI	276 43 57 / 43 26	371 44 62 / 38 26
Jenny *et al* (2020)^10^	Germany, Switzerland, Austria, Belgium, The Netherlands, and France	Cohort	n Age Sex (M / F)% BMI	272 42.9 57.3 / 42.7 26.3	278 44.0 61.5 / 38.5 26.3
Parker *et al* (2013)^11^	Europe	Cohort	n Age Sex (F)% Body weight (kg)	30 38 100 83	16 41 46 81
Adisa *et al* (2018)^12^	Germany	Cohort	n Age Sex (M / F)% BMI	164 46.7 56.8 / 43.2 Not applicable	103 45.7 55.0 / 45.0 Not applicable
Adisa *et al* (2018)^13^	Germany, Switzerland, Austria, Belgium, The Netherlands, and France	Cohort	n Age Sex (M / F)% BMI	267 43 58 / 42 26	103 44 61 / 39 26
Gerrit *et al* (2019) >60^16^	Germany	Cohort (age ≥60 years) Cohort (age <60 years)	n Age Sex (M / F)% BMI n Age Sex (M / F)% BMI Age Sex (M / F)% BMI	38 65.0 63.2 / 36.8 26.7 512 41.9 59.2 / 40.8 26.2 43 57 / 43 26	Not applicable Not applicable Not applicable Not applicable Not applicable Not applicable Not applicable Not applicable 44 62 / 38 26
Aleksandr *et al* (2020)^17^	Russia	Cohort	n Age Sex (M / F)%	126 45 54.9 / 43.3	Not applicable Not applicable Not applicable
Kurzbucha *et al* (2022)^18^	Switzerland	Cohort	BMI n Age Sex (M / F)% BMI	26.7 12 51.6 66.7 / 33.3 25.2	Not applicable 41 55.5 48.8 / 51.2 27.8
Wimar *et al* (2019)^19^	Austria, Belgium, France, Germany, Netherland, Switzerland	Cohort	n Age Sex (M / F)% BMI	272 43 57.4 / 42.6 26	282 44 61.5 / 38.5 26
Scott *et al* (2016)^20^	Not provided	Cohort	n	30	36
			Age	38	41
			Sex (F)%	100	46
			BMI	weight : 83	weight : 81
Ardeshir *et al* (2021)^21^	Not provided	Cohort	n	50	Not applicable
			Age Sex (M / F)%	45.4 46 / 54	Not applicable Not applicable
			BMI	28	Not applicable
Martin *et al* (2013)^22^	Germany, Switzerland, Austria, Belgium, The Netherlands, and France	Cohort	n Age Sex (F) BMI	63 40.5 63 25.9	94 40.5 94 26.6
Ament *et al* (2019)^23^	America	Cohort	n	276	278
			Age	43	44
			Sex (M / F)% BMI	57 / 43 26	62 / 38 26
Vukas *et al* (2013)^24^	Croatia	Cohort	N	30	72
			Age	38.2	40.6
			Sex (M / F)%	53.3 / 46.7	68.1 / 31.9
			BMI	Not applicable	Not applicable
Wimar *et al* (2019)^25^	Not provided	Cohort	n	272	282
			Age	43	44
			Sex (M / F)%	57.4 / 42.6	61.5 / 38.5
			BMI	26	26
Krutko *et al* (2021)^26^	Russia	Cohort	n	133	Not applicable
			Age	38.3	Not applicable
			Sex (M / F)%	54.9 / 451	Not applicable
			BMI	26.7	Not applicable
Adisa *et al* (2020)^27^	Austria, Belgium,	Cohort	n	272	278
	France, Germany,		Age	Not applicable	Not applicable
	the Netherlands,		Sex (M / F)%	Not applicable	Not applicable
	and Switzerland		BMI	Not applicable	Not applicable
Sanginov *et al* (2018)^28^	Russia	Cohort	n	120	Not applicable
			Age	37.6	Not applicable
			Sex (M / F)%	53.3 / 46.7	Not applicable
			BMI	26.6	Not applicable
Brandon *et al* (2019)^29^	America	Cohort	n	75	Not applicable
			Age	Not applicable	Not applicable
			Sex (M / F)% BMI	Not applicable Not applicable	Not applicable Not applicable

The appraisal results of the included full-texts suggest that most of the studies had fair quality (n=13), while there were seven good-quality studies and only two poor-quality studies. The predominance of fair-quality studies suggests methodological limitations in aspects such as sample selection, comparability, or outcome assessment, which could introduce bias and affect the overall reliability of findings. The summary of the NOS score for each cohort study is presented in Table II^4-6,8,10-13,16-29^.

**Table II:** Results from the critical appraisal using NOS.

**Author, (Year)**	**Selection**	**Comparability**	**Outcome**	**Total score**	**Remark**
Pyung *et al* (2019)^4^	★★★	★★	★★★	8	Good
Claudius *et al* (2020)^5^	★★★	★★	★★	7	Good
Michiel *et al* (2012)^6^	★★	★	★★	5	Fair
Adisa *et al* (2017)^8^	★★★	★	★★	6	Good
Peter *et al* (2017)^9^	★★★	★★	★★★	8	Good
Jenny *et al* (2020)^10^	★★	★★	★★	6	Fair
Parker *et al* (2013)^11^	★★	★★	★★	6	Fair
Adisa *et al* (2018)^12^	★★★	★	★★	6	Good
Adisa *et al* (2018)^13^	★★	★★	★★	6	Fair
Gerrit *et al* (2019)^16^	★★★	★	★★	6	Good
Aleksandr *et al* (2020)^17^	★★★	★	★★	6	Good
Kurzbucha *et al* (2022)^18^	★★	★★	★★	6	Fair
Wimar *et al* (2019)^19^	★★★	★★	★★	7	Good
Scott *et al* (2016)^20^	★★	★	★★	4	Fair
Ardeshir *et al* (2021)^21^	★★	★★	★★	6	Fair
Martin *et al* (2013)^22^	★★	★★	★★	6	Fair
Ament *et al* (2019)^23^	★★	★★	★★	6	Fair
Vukas *et al* (2013)^24^	★★	★★	★★	6	Fair
Wimar *et al* (2019)^25^	★★	★★	★★	6	Fair
Krutko *et al* (2021)^26^	★★	★★	★★★	7	Fair
Adisa *et al* (2020)^27^	★★	★★	★★	6	Fair
Sanginov *et al* (2018)^28^	★★	-	★	3	Poor
Brandon *et al* (2019)^29^	★★	-	★★	4	Poor

Notes - Each star (★) represent one score, (-): no score

**Fig. 2:**
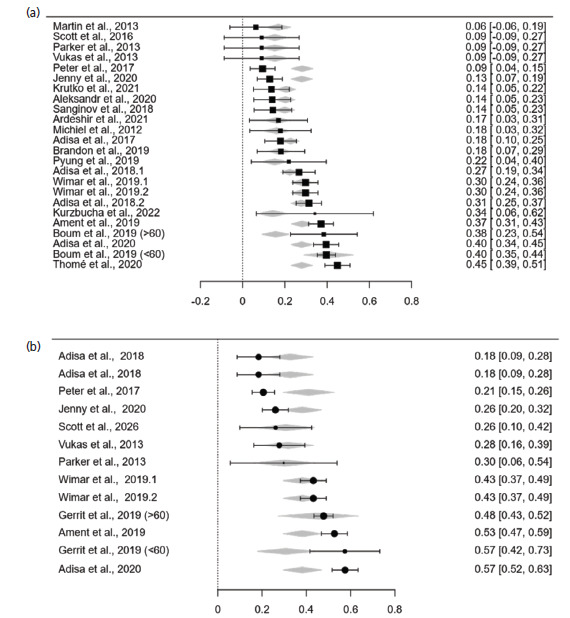
(a) Forest plots for the sample size-adjusted proportion of reherniation incidences in ACD and (b) control.

The prevalence of ACD with and without the sample size adjustment is presented in Table III. The crude prevalence of reherniation in ACD and control groups is 23.2% (95% CI: 18.2%−28.1%) and 36.4% (95% CI: 28.2%−44.5%), respectively. The moderator effect of sample size is significant for pooled data of the ACD group (p-mod=0.002) but not for the control group (p-mod=0.278). After the adjustment with sample size, the prevalences are 13.6% (95% CI: 6.2%−21.1%) and 29.6% (95% CI: 14.9%−33.2%) for the ACD and control groups, respectively. The forest plots for the sample size-adjusted prevalence are presented in Fig. 2. According to Z-statistics, the prevalence of reherniation is significantly higher in the non-ACD group (p-Z<0.001).

**Table III:** Prevalence of ACD before and after the adjustment with sample size.

**Intervention**	**n**	**Prevalence (95% CI)**	**I2 (%)**	**p-Het**	**p-Z**	**p-moderator effect**
Before adjustment ACD	2649	23.2 (18.2−28.1)	88.21	<0.001	<0.001	NA
Control	2546	37.9 (29.7−46.1)	93.76	<0.001		NA
After adjustment						
ACD	2649	13.6 (6.2−21.1)	82.41	<0.001	<0.001	0.002
Control	2546	32.5 (16.9−48.0)	93.63	<0.001		0.417

Note – NA: Not applicable

The statistical significance for the effect of age and BMI as moderators is presented in Table IV. Our model revealed that only age is considered a significant moderator (p<0.001), especially among patients without an ACD implant.

**Table IV:** Moderator effects of age and BMI on the reherniation prevalence.

**Variable**	**ACD**		**Control**	
	**Study, n**	**p-moderator effect**	**Study, n**	**p-moderator effect**
Age	18	0.063	8	<0.001
BMI	18	0.461	5	0.883

**Fig. 3:**
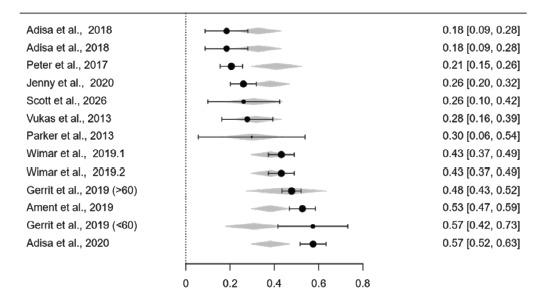
Effect of ACD implant on reherniation risk. OR: 0.433 (95%CI: 0.35-0.53); p-tot<0.001; I2=0%; p-Het=0.461.

There were 11 studies reporting herniation recurrence in patients with and without ACD. The pooled estimate suggests that an ACD implant could reduce the risk of reherniation by 0.433 times (95% CI: 0.35-0.53) with a p-tot <0.001 (Fig. 3). With zero I^2^and p-Het=0.461, the heterogeneity in the pooled estimate is considered negligible.

**Fig. 4:**
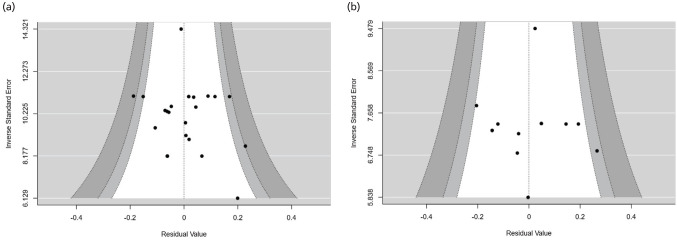
(a) Funnel plot for ACD and (b) control. P-Begg’s of 0.329 and 0.951 are found in ACD and control groups, respectively.

Funnel plots to observe the presence of bias in the reporting of reherniation events are presented in Fig. 4. According to the rank correlation analysis for the asymmetry, no publication bias is detected in both ACD (p-Begg’s=0.329) and control groups (p-Begg’s=0.951).

## DISCUSSION

The primary issue following lumbar discectomy for disc herniation is the risk of reherniation in the post-operative period. Many surgical techniques have been proposed to treat disc reherniation, however, the optimal one remains variable. Our analysis successfully compared the occurrence of symptomatic reherniation in ACD in contrast to lumbar discectomy alone. This meta-analysis demonstrated that incorporating ACD in addition to lumbar discectomy decreased the likelihood of symptomatic reherniation and post-operative complications, as well as the necessity for subsequent surgeries when compared to lumbar discectomy alone.

An implantable bone-anchored device has been developed to lower the chances of symptom recurrence and the necessity for additional surgery in high-risk patients. This aims to ensure long-lasting closure of the annular defect8. Multiple studies have confirmed that following lumbar discectomy with annular closure device (ACD) implantation significantly decreases the risk of re-operation by providing structural support to the annulus fibrosus^8,30^. The ACDs act as a protective barrier that reinforces the weakened area, thereby preventing the inner gel-like core of the disc, known as the nucleus pulposus, from protruding through the defect^8,12^. Usually, after lumbar discectomy, the nucleus pulposus puts pressure on the annulus fibrosus, leading to altered pressure dynamics that cause the nucleus to push through the weakened area. In such cases, ACDs help restore the natural pressure distribution within the disc by sealing the defect, thereby preserving the internal disc mechanics and reducing the risk of the nucleus pulposus migrating and causing reherniation. Also, ACDs provide additional support to reduce stress on the annulus fibrosus, thereby offering extra reinforcement^8,30^. This supplementary mechanical support decreases the likelihood of the annulus fibres tearing again when exposed to physiological loads^8,12^. Additionally, the natural healing process of the annulus fibrosus can be slow and insufficient, especially in the presence of a significant defect^8,12,31^. ACDs create a stable environment that can promote the healing of the annular tissue, by protecting the defect and reducing mechanical stress, they support the body's natural repair processes, leading to stronger and more resilient annular tissue over time^12,30^. In summary, annular closure devices provide mechanical support and stabilisation to the annulus fibrosus after lumbar discectomy, promoting better healing, maintaining intradiscal pressure, disc height, and reducing the mechanical stress on the repaired area^12^. These factors collectively contribute to a lower the risk of reherniation compared to patients who do not receive this additional treatment^8^.

To the best of the author's knowledge, this is the first meta-analysis that investigates the prevalence of symptomatic reherniation following ACD and the contributed factors. A study conducted in 2018 also focused on ACD for disc herniation, but they aimed to evaluate the clinical outcomes and the potential complications^31^. They used four full-text electronic databases that were systematically searched through September 2017. Data including the outcomes of ACD, or annular repair were extracted, and the results were grouped using meta-analysis with weighted mean difference and odds ratio as summary statistics. Using this method, their results were four studies that met the inclusion criteria, of which three studies reported the use of Barricaid (ACD) while one study reported the use of Anulex (AR)^31^. There were 24 symptomatic recurrences reported among 811 operations, compared with 51 among 645 in the control group (OR: 0.34; 95% Cl: 0.20,056; I^2 = 0%; P < 0.0001)^31^. Durotomies were lower among the ACD/AR patients with only 3 reported cases compared to 7 in the control group (OR: 0.54; 95% CI: 0.13, 2.23; I^2 = 11%; P = 0.39). Similar results for the post-operative Oswestry Disability Index and visual analogue scale were obtained when both groups were compared^31^. Their conclusions for the study were as follows: initial results suggest that using the Barricaid and Anulex devices is beneficial for short-term outcomes, demonstrating a reduction in symptomatic disc herniation with the following complication rates Low surgery^31^.

ACD was associated with reduced overall complications, although including reherniations in the complications, category may lead to an inflated complication rate among control patients^10,19^. The results remain inconclusive because of the limited number of events in most studies, and no significant variation in complication rates was noted. However, conducting larger studies with more participants may reveal a discrepancy. The performance bias of operating surgeons might also contribute to the lower complications seen with ACD. ACD results in lower indirect costs by reducing the reherniation prevalence, primarily through reducing re-operations and thus saving long-term costs^11,23^. Several meta-analyses consistently found that female sex, smoker status, and BMI were linked to poor short-term clinical outcomes, while age, disc changes, were not found to impact outcomes^32^, while in our meta-analysis, we found that only age emerged as a significant moderator (p<0.001), particularly among patients without ACD implants. Therefore, there is a need for evaluating ACD in less restrictive “real-world scenarios”.

The current research has limitations that need to be acknowledged. Firstly, data are scarce in the literature regarding this new technology, with only 11 studies being available for analysis. It is essential to conduct additional studies with larger sample sizes and prospective follow-up to validate the findings. The limited availability of studies also led to the inclusion of shorter outcomes (90-day results) in our pooled analysis. Most of the studies had fair quality due to the limitation in the study design which affect the overall quality of the pooled estimates. There was significant heterogeneity in the ACD technology utilised and baseline characteristics, which has been demonstrated to influence disc herniations.

## CONCLUSION

The sample size-adjusted prevalences of reherniation were 13.6% and 32.5% for procedure with and without ACD implant, respectively. Bone-anchored annular closure devices were found to reduce symptomatic reherniation after lumbar discectomy and post-operative complications, as well as the necessity for subsequent surgeries when compared to lumbar discectomy alone.

## CONFLICT OF INTEREST

The authors declare no potential conflict of interest.
